# Assessment of possible impact of a health promotion program in Korea from health risk trends in a longitudinally observed cohort

**DOI:** 10.1186/1478-7954-2-10

**Published:** 2004-11-11

**Authors:** J Park, SH Jee, DW Edington

**Affiliations:** 1 University of Michigan, 1027 E. Huron, Ann Arbor, Michigan 48104-1688, USA; 2 134, Shinchon-Dong, Seodaemun-Gu, Yonsei University, Seoul, Korea

**Keywords:** Health Risk Appraisal, Markov Chain, Health Promotion, Health Status, Risk, Trend, Simulation, Natural Flow, Transition

## Abstract

**Background:**

Longitudinally observed cohort data can be utilized to assess the potential for health promotion and healthcare planning by comparing the estimated risk factor trends of non-intervened with that of intervened. The paper seeks (1) to estimate a natural transition (patterns of movement between states) of health risk state from a Korean cohort data using a Markov model, (2) to derive an effective and necessary health promotion strategy for the population, and (3) to project a possible impact of an intervention program on health status.

**Methods:**

The observed transition of health risk states in a Korean employee cohort was utilized to estimate the natural flow of aggregated health risk states from eight health risk measures using Markov chain models. In addition, a reinforced transition was simulated, given that a health promotion program was implemented for the cohort, to project a possible impact on improvement of health status. An intervened risk transition was obtained based on age, gender, and baseline risk state, adjusted to match with the Korean cohort, from a simulated random sample of a US employee population, where a health intervention was in place.

**Results:**

The estimated natural flow (non-intervened), following Markov chain order 2, showed a decrease in low risk state by 3.1 percentage points in the Korean population while the simulated reinforced transition (intervened) projected an increase in low risk state by 7.5 percentage points. Estimated transitions of risk states demonstrated the necessity of not only the risk reduction but also low risk maintenance.

**Conclusions:**

The frame work of Markov chain efficiently estimated the trend, and captured the tendency in the natural flow. Given only a minimally intense health promotion program, potential risk reduction and low risk maintenance was projected.

## Background

Evidence was found that health promotion programs affect health risks in the US and in many other countries [1-4]. Also, a consistent association of higher risk individuals with higher medical costs implies a potential impact of risk reduction on cost moderation [5,6]. Musich et al. [7] showed that participation in health promotion programs can be effective in moderating medical costs. While most health promotion programs in the US focus on the improvement of health rather than direct economic benefits, many economic evaluations claim that there are transfers of benefits between participation, risk reduction and cost savings [8-12]. However, without identified health risks and a systematic evaluation of the needs to provide the quality programs in the Korean society, an implementation of such programs would be unlikely.

A previous study with a random sample from a 12-year cohort of civil servants in Korea [13] provided an insight that lifestyle factors predicted future medical utilization reasonably well. This suggests that risk status, measured by lifestyle and biometric factors at a point in time, could be used as a pivot to estimate future medical utilization as a result of risk progression.

Longitudinally observed cohort data can be utilized for health promotion and healthcare planning provided the health risk trend is estimated and it poses a general need for an intervention. This paper attempts to address an issue around the possibility of predicting the impact of a health promotion intervention by applying observed effectiveness data from a population with an intervention to the observed transitions of risk status in a Korean population in the absence of such a program.

## Methods

To help in understanding the overall risk transitions in the Korean population and to implement an effective intervention program, a Markov chain model was utilized, assuming finite risk states at any point in time [14]. Today's weather affects tomorrow's but yesterday's may be already irrelevant to tomorrow's forecast. Stock price tomorrow may depend on the previous week's stock prices, not just today's. In estimating the chance of getting "sunny" weather tomorrow, most relevant information to it could be today's weather, where today's weather can be described as "rain", 'cloudy", or "sunny", for example. In general, a random process with fixed number of values could be numerically described, with the collection of possible values forming a "state space" (all possible weather, for example) and the possible values being "states" (rain, cloudy, or sunny, for example). Markov chain models the dependent structure of the future state of a random process on previous states as in the weather forecast example. In the current study, the Markov chain model can describe risk transitions over time when future risk transitions depend on the previous risk states. This modelling is used when a decision problem involves risk state change over time, and interest in the event. This also enables one to project the health status of a population [15-18].

This paper utilizes the observed transitions of measured health risks in a cohort of the Korean National Health Insurance Corporation registrants (KNHIC) over 5 years. This longitudinally-followed population trend, without a particular intervention or policy change in place, was used as a basis to estimate a natural history of risk flow. In addition, an intervened transition was simulated, given that a health promotion program was implemented for the KNHIC cohort to project a possible impact on the improvement of health status. Comparing the two transitions also provided directions for a health promotion program that might be implemented in this population.

### Population

The study population consisted of the established KNHIC prospective cohort [13]. Registrants of KNHIC were invited to complete a health survey prior to each mandatory bi-annual physical examination. The respondents to this preliminary-health risk appraisal (p-HRA) in 1992, were followed bi-annually from 1996 to 2000. Reliability and validation tests were not carried out for the p-HRA. The criteria for inclusion in the study are: (1) actively employed over the period, (2) ages between 30 and 65 in 1996 (N = 180,767).

Similarly, a comparison population was selected for the simulation of a program effect from the large longitudinal database of University of Michigan-Health Risk Appraisal (UM-HRA) completers. The UM-HRA was originally a CDC version, which was modified to fit the national trend of cost, and to meet the guidelines over time. Additional conditions applied are: (1) participated in a health promotion program at a minimal intensity, (2) completed the UM-HRA at least three times during 1996–2000, (3) were insured by the same insurance plans during the corresponding years and (4) were actively employed over the period at the same industry, and (5) age under 65 years in 1996 (N = 15,793).

### Questionnaire

The survey questionnaires (p-HRA), on health status, diet, and lifestyles, were sent to work places and homes to encourage a national health screening at designated health care facilities and to measure lifestyle related health behaviors, every two years for the KNHIC registrants. UM-HRA was used to appraise individual health status during the same period (1996–2000) for the US population. The validity of UM-HRA has been addressed elsewhere [19,20].

### Health risks and costs

Three lifestyle-related health risks were measured by the corresponding questionnaires (p-HRA for the study cohort, and UM-HRA for the comparison population): physical activity, alcohol consumption, and smoking. In addition, pivotal measures of overall health were collected by the questionnaires: perceived health and medical conditions. Three biometric measures were obtained from the appropriate lab tests during physical examination for the KNHIC population and from the self-reported measures for the comparison population: systolic blood pressure (SBP) /diastolic blood pressure (DBP), total cholesterol, and body mass index (BMI) via height and weight measures. The risk criteria for the US population were defined (Table 1) following the published guideline by the US–CDC/Carter Center, and some were modified to fit better for prediction of healthcare costs (BMI and physical activity).

**Table 1:** Risk Evaluation Criteria and baseline characteristics. Individuals from US population were classified as Low, Medium, and High risk as in KNHIC population. Within each risk group, random samples were selected each time of sampling from US population stratified with age and gender once they met the similar risk profile of KNHIC. This bootstrap-sampled match would be used as a control (interven ed) population.

*Baseline characteristics*	*KNHIC population (N = 180,767)*		Comparative population with intervention (N = 180,767)*	
*Risks*	*Criteria*	*N (%)*	*Criteria*	*N (%)*

Perceived Health	Poor /Fair	24,290(13.4)	Poor /Fair	31,814(17.6)
Exercise	Less than 1/week	100,395(55.5)	Less than 1/week	56,399(31.2)
Alcohol	Drink>7/week^1^	17,554(9.7)	Drink>14/week	16,630(9.2)
Smoking	Current smoker	55,052(30.4)	Current smoker	29,645(16.4)
BMI	BMI>25.0 for male, >23.0 for female^2^	46,227(25.6)	BMI>27.50	69,776(38.6)
BP	SBP> 120 or DBP>80^3^	91,924(63.9)	SBP> 139 or DBP>89	48,084(26.6)
Cholesterol	Cholesterol>220^4^	32,118(17.2)	Cholesterol>239	7,954(4.4)
Medical condition	Self-reported disease	9,280(5.1)	Self-reported disease^5^	35,973(19.9)
Baseline Class	Average Age = 40.0	Male (61%)	Average Age = 40.0	Male (61%)

Low Risk (0–2)		62.9%		63.0%
Medium Risk (3)		22.2%		22.3%
High Risk (4+)		14.9%		14.7%

Note

^1^a drink of "Soju" = 2 drinks of wine/beer

^2^WHO guideline (1999) = 23.5 for Asians

^3,4^Korean Medical Association (2000) guideline

^5 ^diabetes, heart problem, cancer, past stroke, bronchitis/emphysema

*Simulated data after adjusted for age, gender, and baseline risk for KNHIC population

Information on eight health risks for the study cohort were systematically evaluated and mapped to the measured risks by UM-HRA (Table 1). This was done according to: (1) the published guidelines for Asians, (2) empirical comparison of question by question, and (3) age and gender adjusted association to the respective healthcare costs distribution [13,21-25]. In addition, different risk criteria were applied to alcohol consumption and medical condition due to systematic difference in measurement. Corresponding health states per period were defined according to the distribution of the aggregated risk state (sum of individual risks variable states) as low (0–2 risks), medium (3 risks) and high (4+ risks).

Inpatient plus outpatient costs per annum were collected from KNHIC database for the medical utilization in association with health risks, and the inflation adjusted average 1996 costs (January 1st, 1996 through December 31, 1996) were used for the T1 costs. Similarly, average T2 costs were calculated from 1998 claims costs.

### Program

Participants of the p-HRA were not given any further information on identified health risks. Neither was it used to gain access to any health intervention programs during the five years (1996–2000). In US, on the other hand, as part of an intervention program, the completers of UM-HRA were given individually tailored health information, followed by encouragement of participation in a health promotion program at no cost during 1996–2000. This nation-wide program included an annual mail-based HRA, personalized follow-up report, identification of top significant risks and referral to health resources. This was defined as minimal level intervention, which differs from KNHIC's p-HRA in providing health information and individual feedback.

### Trend

Provided that the least resources were available for a health intervention of the KNHIC population, a projected health risk transition with the minimal level intervention was utilized for the simulation of possible impact on risk transition. Population health trends were followed over the three time frames (T1, T2, and T3). T1 refers to the baseline year, which is 1996 for both populations. T2 refers to the second time frame, which is 1998 for the KNHIC population, and 1997 or 1998 for the compared US population. T3 refers to the 3^rd ^time frame, 2000 for the KNHIC population, and 1998 or 2000 for the US population. Population with minimal intervention was matched to the baseline characteristics of KNHIC population, using age, gender, and baseline risk distribution. Trend was defined as the risk state change in each population between the time-points while each transition was annualized. Each change of risk state was estimated and the parameters to trend from the matched intervened population were used to project the possible trend of KNHIC population, following such a program.

### Analysis

An age/gender-cohort model was implemented, following observations on the natural flow of health status over three time frames, and it was compared to the corresponding age/gender-cohort of a US population with the matched risk distributions, where an intervention at minimal level was applied.

For a simulation of an intervened transition for KNHIC population, an adjusted estimation of intervened transition was obtained based on random samples drawn from the selected US sample of intervened employees (N = 15, 793). To simulate additional random samples to reduce variances in the estimate of parameters for transition, a Monte Carlo bootstrap [26,27] method was employed to the sub-populations with age, gender, and baseline risk matched to the KNHIC populations (N = 180, 767). For each 10 years apart (30–40, 41–50, 51–60) cohort from the matched boosted random samples of US population, risk transitions following a minimal intervention were estimated while controlling for age, gender, current and previous risk states, and previous year's medical claims costs. Similarly, natural flow of risk transitions was estimated from the observed KNHIC cohort data. The estimated parameters (covariate and baseline effect, corresponding mean, variance and covariance) and fitted model for the intervened transition was applied to the KNHIC population for the transition probabilities for each risk state at T3. Finally, an aggregated weighted probability for transition was calculated based on age-gender-risk state distribution.

**Figure 1:**
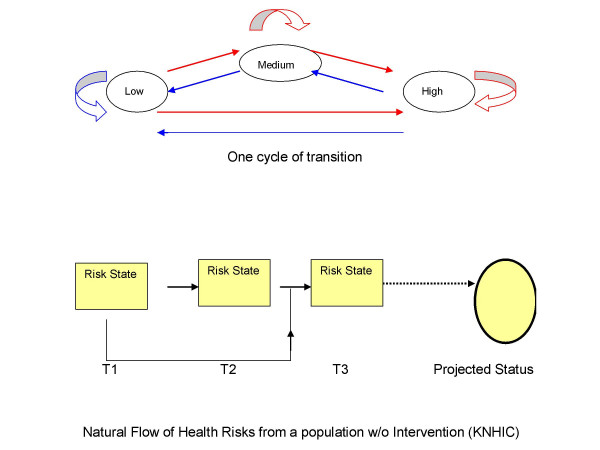
Markov transition with 3 risk states without an exit.

Computations were carried out using a generalized linear model for the ordered categorical outcomes (low risk<medium risk<high risk), assuming a Markov chain model in which the order of dependency was learnt from the data (Figure 1). Figure 1 depicts the transition between risk states at each cycle and the dependency of risk progress on the previous risk state. All risk states are inter-connected and allow feedback cycles at each risk state (staying at the same state).

Assumptions of Markov chain model, and model fit diagnostics were evaluated for the KNHIC population. Stationarity was tested by likelihood ratio test of the fitted models using a generalized equation estimation with and without time-dependence while controlling for other measured covariates. Order of Markov chain was tested with likelihood ratio test and fit-diagnostics were run. Following the fitted Markov model, we presented the estimated transitions over three time-frames for the natural flow. Cochran-Armitage trend tests controlling for T1 or T2 risk state were performed to test the trend of dependency of future state on T2 or T1 risk state.

Average costs in each T1-T2 risk state pair were calculated from the corresponding years' average costs, adjusted for inflation. Healthcare costs were converted to ratios of average costs per T1-T2 risk transition state, compared to low (T1)-low (T2). Adjusting with any appropriate inflation rate in the future, these ratios can be used to project the cost savings over time by comparing the number of projected trends times cost ratios in natural and intervened flows. The projected percentage changes in the two trends (natural vs. intervened) were compared for an estimation of an intervention impact on risk change.

## Results

### Feasibility of Markov chain model

Individual baseline risk prevalence of the two cohorts is shown in Table 1. The simulated intervened cohort was adjusted for age, gender, and baseline risk states according to those of KNHIC data. Compared to the US population, lack of physical exercise, and smoking were significantly more prevalent among the Korean cohort at baseline. Biometric risks such as blood pressure and cholesterol were higher in the KNHIC population due to 100% compliance rate of lab test of the study population. Overweight and medical condition was significantly less among the Korean. However, overall baseline risk distributions based on the clustered state (low, medium or high) were about the same. A simulation of an intervention effect follows the adjusted baseline characteristics of the comparative study population and Table 1 shows similarity of the two populations at baseline such as age, gender and risk distribution for comparability.

Under the assumption of finite number of health risk states (low, medium and high states), Markovian models were examined for proper order estimation, an association structure, and stationarity. Following a likelihood ratio test, Markov chain (MC) order 2 was preferred for the risk transition of the non-intervened (KNHIC) population (Table 2). In other words, future state depends on the current and the most recent past states and when controlled for the dependency on risk state, overall future risk state depends on the individual risk factors far less significantly. This also assured that matching intervened trend is applicable to the KNHIC population without further concern on effect of inherent risk progress to future risk transition. Therefore, for the following estimations of transitions, the dependency specified in Table 2 was used.

**Table 2:** MC order test using cumulative logit model of becoming low risk at T3

*Selected Predictors (Significant, P < 0.01)*	*MC order = 1*	*MC order = 2*
Male	-0.563	-0.345
Age	-0.023	-0.019
Baseline risk (t1) Low	2.73	1.545
Baseline cost (t1)	-0.01	-0.011
Risk at t2 Low	-	1.938
Model fit		
log L	-283085	-139246*

* Log likelihood ratio test of order 2 model, compared to order 1 model (287,678 >> χ^2^(2), pr<0.001) was significantly preferred.

### Characteristics of the obtained trend

When Markov chain order 2 was assumed, higher T1 risk status, controlling for T2 risk state as in Table 3, was associated with lower percentage of being at low or lower risk state at T3 (T3 state = "0" if at low or lower state than the state at T2. T3 state = "1" if at high or higher state than the state at T2, Pr<0.001). Similarly, higher T2 risk state, controlling for T1 risk, was less likely to be at "0" at T3 (Pr<0.001). Due to a stronger dependency on T2 risk state, medium (T1)-low (T2) is more likely to be at low at T3 than low (T1)-medium (T2). However, additional dependency on the T1 risk state differentiates the trend percentages at "0" state (T3) from medium (T1)-low (T2) and high (T1)-low (T2).

**Table 3:** T1-T2 risk state by T3 risk state. Note that outcome is 0 if T3 state is low or lower than the state at T2, otherwise it is 1. The test for trend controlling for T1 risk state is: a>b>c; d>e>f; g>h>i (Pr>z<0.001). The test for trend controlling for T2 risk state is: a>d>g; b>e>h(Pr>z<0.001)c>f>i (Pr = 0.143) The superscripted numbers in parenthesis represent the order of trend which appeared to be significantly associated with the likelihood to be at "0" at T3.

	*Trend percentage at T3 per T1-T2 risk state*			
*T1-T2 risk state *(N = 180,767)	*(0)*	*(1)*	*Comparison of order*	*Trend statistic (Z)*
Low-Low^a^	82.4^(1)^	17.6	Vs. d	-68.2*
Low-Medium^b^	52.5^(3)^	47.5	Vs. g	-11.4*
Low-High^c^	35.8^(5)^	64.2	Vs. e	-44.7*
Medium-Low^d^	58.6^(2)^	41.4	Vs. b	-11.2*
Medium-Medium^e^	34.0^(6)^	66.0	Vs. h	-18.0*
Medium-High^f^	21.5^(8)^	78.5	Vs. i	-27.7*
High-Low^g^	43.5^(4)^	56.5	Vs. c	-26.2*
High-Medium^h^	22.6^(7)^	77.4	Vs. f	-43.8*
High-High^i^	11.7^(9)^	88.3	-	-

* Significant with α = 0.01

Cochran-Armitage trend tests controlling for T1 risk state or T2 risk state show a strong declining trend of staying at "0" than that at T2 as in the order of appearance in Table 3. For example, low (T1)-low (T2) is more likely to be at low in T3, compared to low-medium and low-high. This tendency holds for: a>b>c; d>e>f; g>h>i with Pr<0.001, a>d>g; b>e>h with Pr<0.001 except c>f>i (Pr<z = 0.143), where a, b, c; d, e, f; g, h, and i corresponds to low-low, low-medium, low-high; medium-low, medium-medium, medium-high; high-low, high-medium, and high-high risk state, respectively. Also, overall consistency appears significant (one-sided, Pr <z: <0.001). Pairwise comparisons determined the order of likelihood to be at low or lower state at T3 (i.e. outcome = 0) in association with T1-T2 risk state. Low (T1)-low (T2) tops the order, with 82.4 % of the individuals being at low risk at T3, followed by medium (T1)-low (T2) with 58.6%, low (T1)-medium (T2) with 52.5% and so on (a>d>b>g>c>e>h>f>i, Pr<0.001).

### Application of Markov chain order 2 for estimation of natural and reinforced transition flows

We applied the results from the previous sections (regarding the KNHIC population) to the observed risk transitions of the matched sample from a US active employee population, who participated in a minimal level program. Similar assumptions were tested and stationary Markov chain order 2 was also applied. The estimated transition probabilities without an intervention (KNHIC cohort) and those of the same people with a minimal level intervention are shown in Table 4. The higher probabilities to be at low risk state at T3 were shown in the intervened flow (except medium (T1)-high (T2) and high-low) compared to the natural flow.

**Table 4:** Estimated risk transition probability with and without intervention and medical utilization.

	*Behavioral health risk measured at two times (T1 and T2)*	*T1-T2 Utilization Cost Ratio^b^*				*KNHIC population without intervention*			*KN HIC population with a simulated intervention at minimal level^a^*		
			
			***Risk state at T3***			***Risk state at T3***		
		
T1	T2		*Low*	Medium	*High*	*Low*	*Medium*	*High*
Low	Low	1.00	0.81	0.15	0.04	0.97	0.03	0.00^c^
	Medium	1.14	0.53	0.34	0.13	0.69	0.11	0.20
	High	1.58	0.38	0.32	0.30	0.65	0.34	0.01
Medium	Low	1.16	0.60	0.30	0.10	0.65	0.33	0.02
	Medium	1.23	0.35	0.44	0.21	0.39	0.32	0.29
	High	1.52	0.23	0.35	0.42	0.05	0.73	0.22
High	Low	1.51	0.46	0.31	0.23	0.34	0.65	0.01
	Medium	1.52	0.25	0.38	0.37	0.34	0.65	0.01
	High	1.79	0.14	0.27	0.59	0.16	0.30	0.54

^a^Risk transition probability was estimated using Markov model order 2, adjusted for age and gender distribution of KNHIC.

^*b*^Comparing average cost in each T1-T2 risk state pair (e.g. high-medium) to that of the low-low (T1-T2).

^*c*^All probabilities were rounded off at the 3^rd ^decimal place (i.e.0.0014).

Overall, the likelihood of maintaining health at low risk (T1-T2-T3) is higher in the intervened transition (0.97 vs. 0.81). Also, the probabilities of being at high (T1-T2-T3) and at medium (T1-T2-T3) are lower in the intervened flow (0.54 vs. 0.59 at high, 0.32 vs. 0.44 at medium). Healthcare cost ratios in relation to the T1 -T2 risk states in the KNHIC population are also shown in the table. In general, cost ratios increased in the order of T1-T2 risk states and cost utilization of high-high group almost doubled (1.79) the cost of low-low.

After three years of projection, the transitions, presented in Table 5 show the estimated numbers in each risk state, following the natural and the intervened flows as in Table 4. Three-year forward projection of populations (non-intervened vs. intervened) was calculated by pre-multiplying the number of people in each T1-T2 risk group to the 3^rd ^power of the Markov transition matrix of order 2. This is then collapsed by the baseline states as in Table 5 to show the net gain from T1 to T3. The difference was calculated as the projected counts per state from the baseline total counts, and percentage point changes were calculated (Table 6). Overall, there was 0.72% point net increase in high risk state following from natural flow and 5.01% points net decrease in the intervened flow. Low risk percentage decreased by 3.07% points following the natural flow but increased by 7.49% points in the intervened flow.

**Table 5:** Projection of population KNHIC following natural vs. intervened flow over 3 waves. Projected 3 – forward years based on MC-order2

*Risk state*	*At Baseline*	*At T3*^α^	*At T3*^β^
Low	113,605	108,050	127,149
Medium	40,151	44,400	35,675
High	27,011	28,317	17,943

**Table 6:** Projection of population KNHIC following natural vs. intervened flow over 3 waves. Percentage Point change following Table 4-(a)

*Risk state*	*At Baseline*	*At T3*^α^	*At T3*^β^
Low	113,605	-3.07%	+7.49%
Medium	40,151	+2.35%	-2.47%
High	27,011	+0.72%	-5.01%

^α,^Following natural risk flow, ^β^Following intervened flow

## Discussion

### Markov Chain Model and Transitions of Risk States

After controlling for age, gender and other covariates, such as past healthcare costs, variations within groups in predicting the future risk transition were left unexplained. This suggested an additional consideration of dependence on other factors such as past health risk history. This dependent structure of a natural risk flow was best fitted with a Markov model order 2 (due to limitation of the data, no higher order was tested). In other words, current risk states largely depend on the immediate past state and also depend on the one before that. The estimated probability was stable. There was no policy change or environmental effect, which is considered as a period effect, during the study period. In addition, as a result of testing a higher order MC for the available US data, order 2 was preferred to the order 3 (data not shown). Therefore, we concluded that an assumption of MC order 2 was plausible for the health risk transitions regardless of natural flow or reinforced flow at minimal intensity [28,29]. Also, although the time-frames of observations in the two population were different, time of observation turned out to be non-influential to the transition (stationary) and average years of observation was used to calculate an annualized transition for both transitions. Therefore, the difference in observed years would not have implications for the results.

### Difference in Individual Risk Profile

Simulated data for a minimal program participation, adjusted to matched age, gender, and baseline risk distribution of KNHIC population for comparison, showed that sampling disparities were significantly reduced in overall distribution and demographics but still remained in individual risk factors. Exercise and smoking risks are relevant to environmental and cultural adaptation, which showed large differences in prevalence. Typically, overweight is the most prevalent risk factor in the US, and it appeared to be significantly higher even after matching overall risk distribution to the Korean population. Although there exist some differences, these biases in the data were assumed to be smoothed out by collapsing to the aggregated risk levels.

There may be inherent differences in risk transitions in the two populations due to two factors: (1) disparities in the individual risk factors, (2) systematic differences due to cultural and environmental factors. Due to the fact that estimation of risk transition was primarily dependent on the aggregated risk state rather than the individual risks (Table 2), the potential influences were minimal. Matching on individual risk factors additionally may reduce such a bias in the transition. However, individual risk profiles are inconsistent at times and risk transitions depend more on inter-associations of individual risk profiles. Therefore, matching on individual risk factor and transitions based on such profiles may even create another type of bias in the estimation of transition. On the other hand, by bootstrapping of random samples, matching on age, gender, and risk distribution, variations within each risk state have been reduced and even reduced the potential bias due to disparity in individual risk factors. The remaining disparity in the matched populations would become irrelevant when additional control for those remaining disparity factors is made in the model for the ordered categorical transition.

### Implementing a program based on found dependent structure of risk transition

Without reinforcement, the male population was less likely to be in the lower risk states in future compared to the females from the same past (T1 and T2) risk state, given age (Table 2). This suggested an observed potential for improvement of men's health such as an emphasis on the cultural adaptation of health by changing organizations and communities to create supportive environment and re-orienting health services [30].

Aging has been found to be related to the risk transition because as one gets older, he/she is less likely to be in lower risk state in future without intervention (Table 2). There have been concerns about the elderly population, who are under-represented due to unequal resources, and less interest [31,32]. Studies in the US found that current elderly population is healthier than the elderly 10 years ago [31-33]. The observed trend among the elderly may be linked by the changes in health-related individual behaviors in the past. This indicates the potential impact of continuous health promotion on aging adults.

Although the immediate past risk states showed the highest importance, when they are the same, risk state at T1 plays a key role in predicting T3 risk state. In other words, individuals currently at a state may have different paths to the current risk state and the weight (by intensity or allocation of resources) of risk reduction program should differentiate this path-variability to maximize the impact (Tables 3,4). Following the natural flow, the likelihood of those at low at T2 to be at the same low in future (T3) is significantly less than that of those who were on intervention, regardless of their risk state at T1. This suggests a continuing effort on those who once impacted, to maintain their modified health practices until they adapt to their newly improved lifestyle (Table 4).

Over the period, although there was presumably an aging process in the natural flow, there exists a regression to the previous state. Especially with Markov chain order 2, in controlling for the T1 state, the tendency stays. This implies that even without any external reinforcement for a healthier state, low risk state largely tends to maintain its state, followed by the high risk state. Therefore, an intervention with minimal effort to sustain healthy lifestyle and behavior for those who are at low risk may yield substantial benefit in the long run (Tables 3,4). Likewise, an intervention program to break high-high cycle to lower the health risks and eventually to optimize the utilization of the health resources is anticipated to be effective and necessary. These, in association with the cost trend following the risk trend (Table 4), imply that moderation of healthcare costs is also achievable by a well-targeted health promotion program.

### Projected effect of a minimal level intervention

For this study population, the minimal level intervention appeared to be effective in the low and medium baseline groups (Table 4). This is reasoned that the particular program, which was designed to impact people with minimal resources, turned out to be most effective on "low risk maintenance". Thus, efforts to reduce risks were rather undersized. However, people at the high baseline risk except high (T1)-high (T2), were less likely to stay at high risk (at T3) than the non-intervened, showing a potential improvement in risk reduction as well. This was found in similar contexts and quite consistent across studies [34,35]. As a conclusion, these findings suggest that with limited resources, a program that delivers an individual feedback upon completion of an HRA, identifying most significant risks and referring to available resources, could have an impact on the natural health risk flow, especially on low risk maintenance by enhancing awareness and self-efficacy in maintaining good health practice.

One of the study goals was to achieve estimate a natural flow of the study population, where a demand and necessity for health promotion program has been increased recently. Natural flow was coined by Edington [14] to describe a health trend in a population without any intervention for a reinforced effort to improve heath state. By comparing it to various simulated trends with an intervention to the population, one could assess program effectiveness in terms of health change, which was measured as the risk state. This again, with cost-ratios per risk transition as in Table 4, may be used to calculate the possible savings in association with any program of interest. For example, comparing to low-low group, low-high incurred 158% of the costs of low-low. Trend at low at T3 following the intervention was increased by 16% from low-low, comparing to natural flow. Therefore, the potentially incurred costs due to increased risk state could be saved proportionately by 14% (1.14:1.00, 12% of the low (T1)-low (T2) avoided transition to medium (T3)), 58% (1.58:1.00, 4% avoided transition to high (T3)) of the costs. This concept was used wherever health behavior change and lifestyle modification is applicable in an effort to promote and manage health. Thus, health trends following such a program could be compared to the expected natural flow for a demonstration of "effectiveness". In the current study, the estimated natural flow shows decreases in low risk state by 3.07 % whilst 7.49 % increases by following an intervention. The underlying risk progression may be likely explained elsewhere [37].

### Recommendation

It is recommended to set a policy and allocate resources, tailored to the population profile of aggregated risk state, first. Targeting risk factors in the context of risk state or risk clusters could follow next. For example, an overweight population at high risk state requires more resources and more intensive interventions than the overweight population at low risk state. The study provides the base information when planning such a population-based intervention. This approach differentiates from those interventions targeting individual risk profiles, which could be inconsistent at times. Studies suggest that an inter-association over risks is more important in implementing an intervention than individual risks [38]. Some variables are highly variable such as medical conditions, depending on genetic traits more than the environments. This variation may indicate that the level of a risk factor may not be acceptable in one population while it was so in another. This varying manifestation could have an influence on implementing interventions targeting on particular risk factor, which is beyond the scope of the paper.

## 5. Conclusions

As a reasonable method to project the risk trends, a stationary Markov model of order 2 was fitted for the health risk transitions of the non-intervened population, suggesting a natural risk flow for the population. Utilizing this with the matched intervened population, the reinforced risk transitions of the KNHIC population were estimated. The significant difference in the transitions appeared to be for the low baseline risk population even with a minimal intensity intervention program. Therefore, the difference in the projected numbers by the two transitions (with /without interventions) showed a significant impact on low risk maintenance although higher risk population was also impacted to increase fewer risks and to moderate it to becoming "medium risk".

Conventionally, most programs in the past have focused on risk reduction although low risk maintenance has been raised as a practical issue. Since this paper suggests a strong dependence on the previous history of risk status and the instability of high (T1)-low (T2) risk population, compared to low-to-low or medium-to-low population, differentiating efforts for the low risk people (from low to low) and for those moved to low (from high to low), should be sought for maximum impact.

The studied intervention with minimal intensity was to diversify the beneficiaries of the program, to increase awareness, to continue educating and motivating individuals to adopt healthier behaviors by individualized feedback, and to provide resources [36]. The findings demonstrated that even such a minimally intense program could be effective in moderating health risks, preventing relapse and sustaining healthy behaviors over time.

### Limitation and future research

Population characteristics on risk transition were assumed to be similar by adjusting for age, gender, if previous risk states were the same. However, in addition to the measured risks, diet and culturally inherited behavioral differences could make an inherent transitional difference in the two compared populations. Also, some risks such as disease were underestimated in the KNHIC population. Therefore, the presented natural flow estimation in Korean population can be adopted but utilized with caution.

It may be conjectured that there are some risk factors are more easily modifiable than others such as exercise while medical condition and overweight may not. The currently shown disparity in the two compared risk profiles may have induced somewhat less significant program impact on risk transition (lowering risk state) due to higher prevalence in medical conditions and overweight in the control population. This study tried to match two populations as close as possible and to model to reduce potential biases but comparing two populations requires more caution in further relevant studies.

Despite the fact that individual risk distribution is not consistent often time, not only an overall health status but a multivariate risk state (i.e. a matrix per person) could be utilized to identify an effect of each risk factor to their projected state in future, as in Manton and Stallard [37]. Based on the estimated transitions, prediction of the effect of different intervention models on the risk transitions and the impact on healthcare costs would be available. Validity of the p-HRA (KNHIC) was not tested although it was matched to and tested by the established UM-HRA. This will be validated and tested against UM-HRA upon availability of cohort data, which were followed longer time. Markov higher order model (3 +) with a longer follow-up time could be explored for exact consistency and stationarity for the cohort. Even the progression rate from no incidence state to symptomatic state utilizing the health care cost database can be added to the cohort to project the proper care of medical conditions. Inclusion of an "exit" state (such as death) and consideration of a reasonable compliance rate would possibly make the model robust over the longer time of an intervention. We will further investigate the possibility of matching individuals in the two compared populations across many covariates including individual risk factors and other confounders such as willingness to improve, to further reduce potential biases.

## Competing interests

The authors declare that they have no competing interests.

## Authors' Contributions

JP carried out the analyses and drafted the manuscript. SHJ collected, cleaned the data and provided initial analyses. DWE participated in the design of the analyses and provided revisions. All authors read and approved the final manuscript.
